# Identifying potential biomarkers for non-obstructive azoospermia using WGCNA and machine learning algorithms

**DOI:** 10.3389/fendo.2023.1108616

**Published:** 2023-10-03

**Authors:** Qizhen Tang, Quanxin Su, Letian Wei, Kenan Wang, Tao Jiang

**Affiliations:** ^1^ Department of Urology, The First Affiliated Hospital of Dalian Medical University, Dalian, Liaoning, China; ^2^ Department of Andrology, The Second Affiliated Hospital of Dalian Medical University, Dalian, Liaoning, China

**Keywords:** non-obstructive azoospermia, biomarker, machine learning, WGCNA, immune infiltration, diagnosis

## Abstract

**Objective:**

The cause and mechanism of non-obstructive azoospermia (NOA) is complicated; therefore, an effective therapy strategy is yet to be developed. This study aimed to analyse the pathogenesis of NOA at the molecular biological level and to identify the core regulatory genes, which could be utilised as potential biomarkers.

**Methods:**

Three NOA microarray datasets (GSE45885, GSE108886, and GSE145467) were collected from the GEO database and merged into training sets; a further dataset (GSE45887) was then defined as the validation set. Differential gene analysis, consensus cluster analysis, and WGCNA were used to identify preliminary signature genes; then, enrichment analysis was applied to these previously screened signature genes. Next, 4 machine learning algorithms (RF, SVM, GLM, and XGB) were used to detect potential biomarkers that are most closely associated with NOA. Finally, a diagnostic model was constructed from these potential biomarkers and visualised as a nomogram. The differential expression and predictive reliability of the biomarkers were confirmed using the validation set. Furthermore, the competing endogenous RNA network was constructed to identify the regulatory mechanisms of potential biomarkers; further, the CIBERSORT algorithm was used to calculate immune infiltration status among the samples.

**Results:**

A total of 215 differentially expressed genes (DEGs) were identified between NOA and control groups (27 upregulated and 188 downregulated genes). The WGCNA results identified 1123 genes in the MEblue module as target genes that are highly correlated with NOA positivity. The NOA samples were divided into 2 clusters using consensus clustering; further, 1027 genes in the MEblue module, which were screened by WGCNA, were considered to be target genes that are highly correlated with NOA classification. The 129 overlapping genes were then established as signature genes. The XGB algorithm that had the maximum AUC value (AUC=0.946) and the minimum residual value was used to further screen the signature genes. IL20RB, C9orf117, HILS1, PAOX, and DZIP1 were identified as potential NOA biomarkers. This 5 biomarker model had the highest AUC value, of up to 0.982, compared to other single biomarker models; additionally, the results of this biomarker model were verified in the validation set.

**Conclusions:**

As IL20RB, C9orf117, HILS1, PAOX, and DZIP1 have been determined to possess the strongest association with NOA, these five genes could be used as potential therapeutic targets for NOA patients. Furthermore, the model constructed using these five genes, which possessed the highest diagnostic accuracy, may be an effective biomarker model that warrants further experimental validation.

## Introduction

The World Health Organisation estimates that approximately 10–15% of couples in the world currently experience infertility, with males accounting for approximately half of all infertility aetiologies (1, 2). Azoospermia refers to the absence of spermatozoa in the semen, and accounts for 20% of total male infertility cases (3). Clinically, azoospermia can be divided into obstructive azoospermia (OA) and non-obstructive azoospermia (NOA). Specifically, OA refers to cases in which there is obstruction of the distal seminal duct but normal testicular spermatogenic function; in contrast, NOA refers to patients with testicular dysfunction, abnormal spermatogenic function, and inability to produce sperm. Approximately 60% of azoospermic patients are eventually diagnosed with NOA, which is one of the most serious forms of male infertility; however, the aetiology for NOA remains unclear (4, 5). Therefore, it is necessary to elucidate the molecular mechanism of spermatogenesis and to identify effective diagnostic markers or therapeutic targets for NOA.

The continued development of second-generation sequencing technology continuously assists in improving our understanding of the onset and development of diseases at the genetic level. A few previous studies have explored potential biomarkers involved in NOA occurrence (6–9). Most of these studies have diagnosed NOA by a single marker. In addition, these studies lacked the exploration of the mechanisms of marker regulation. The construction of a disease-associated competitive endogenous RNA regulatory network could help to analyse the biological mechanisms of key genes in disease regulation. And most of them lacked the analysis of immune infiltration, where the immune system plays an important role in spermatogenesis (10). These omissions set the stage for the present study.

**Figure 1:**
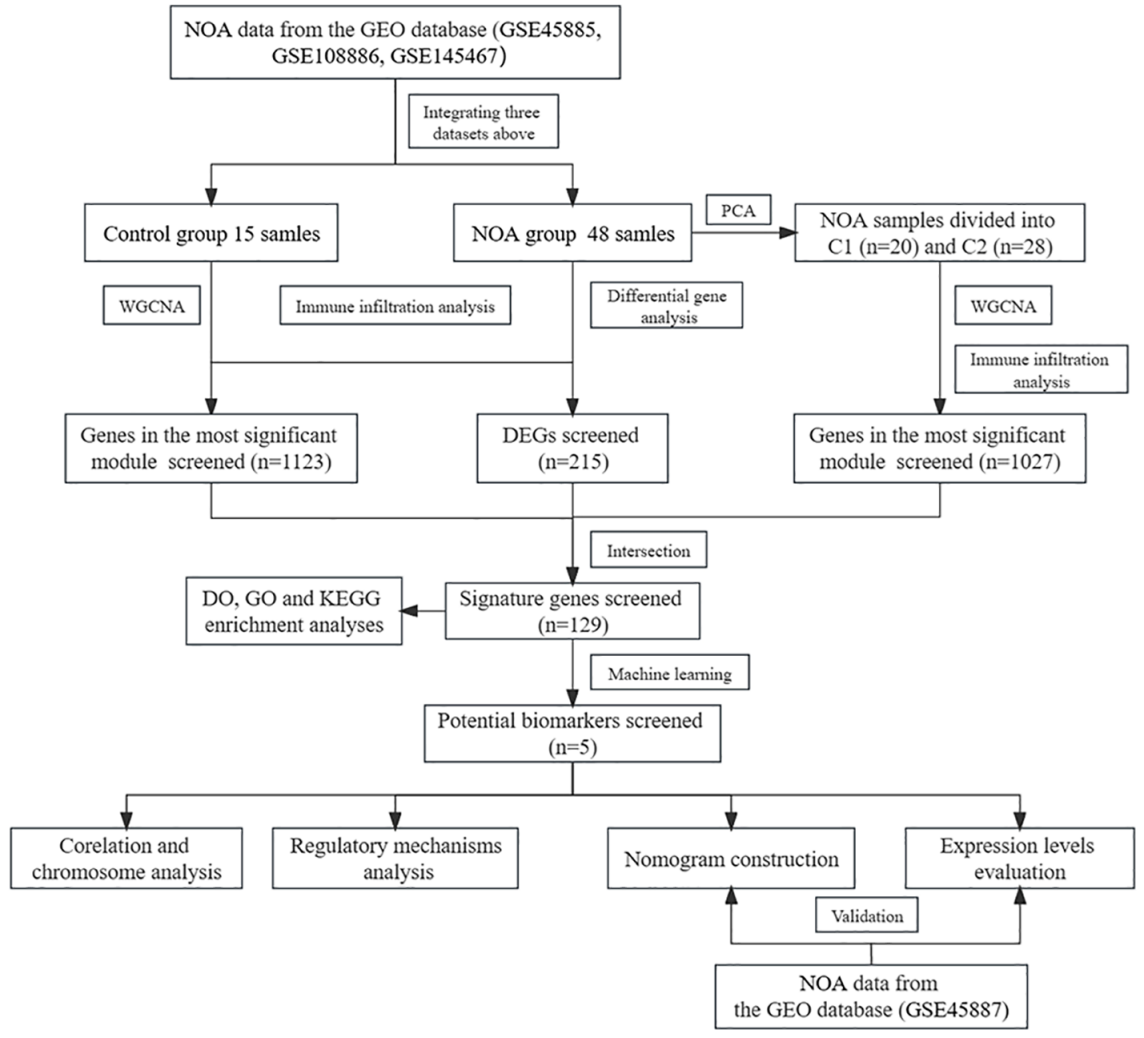
The workflow chart of data processing, analysis and validation. (The gene expression matrices of GSE45885, GSE108886, and GSE145467 were combined and defined the new matrix as the training set; the GSE45887 was defined as the validation set. In the training set, PCA, WGCNA, difference analysis and machine learning algorithms were used to screen potential biomarkers of NOA. Diagnostic models were constructed based on the potential biomarkers and validated using the validation set. Finally, immune infiltration analysis, regulatory analysis and chromosome analysis were performed).

In this study, according to the workflow chart shown in **Figure 1**, we performed systems biology analysis of azoospermia using the GEO database. To identify potential biomarkers and therapeutic targets for the clinical treatment of male infertility, we compared the expression profile data of testicular tissues from NOA patients and normal spermatogenic men; additionally, we screened the key NOA-associated genes using differential gene analysis, consensus cluster analysis, Weighted gene co-expression network analysis (WGCNA) (11), and machine learning algorithms. We also calculated the infiltration of different immune cell populations within NOA patients and analysed the correlation between the hub gene and immune cells. Finally, the competing endogenous RNA network and the diagnostic model we constructed in this study provided a robust basis for further research.

## Methods

### 2.1 Acquiring and processing datasets

We searched for NOA-related raw data in the GEO database (https://www.ncbi.nlm.nih.gov/geo/). Finally, we downloaded four datasets that assessed testes tissue from patients and controls: GSE45885 (controls, 4; NOA patients, 27), GSE45887 (controls, 4; NOA patients, 16), GSE108886 (controls, 1; NOA patients, 11), and GSE145467 (controls, 10; NOA patients, 10) (**Table 1**).

**Table 1:** GEO datasets used in the study.

GSE series	Type	Sample size		Platform	Category
		Control	NOA		
GSE45885	mRNA	4	27	GPL6244	Training set
GSE45887	mRNA	4	16	GPL6244	Validation set
GSE108886	mRNA	1	11	GPL10558	Training set
GSE145467	mRNA	10	10	GPL4133	Training set

**Figure 2:**
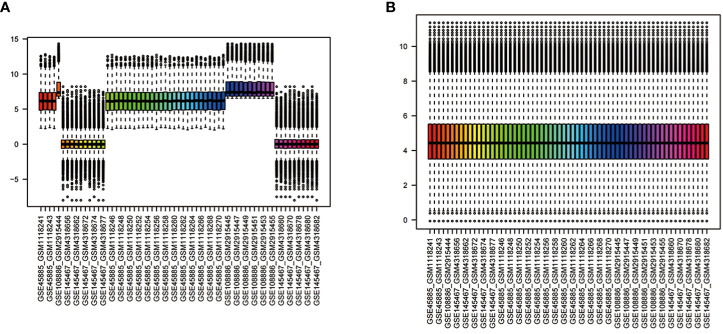
Normalisation of gene expression data in samples **(A)**. Before normalisation **(B)**. After normalisation.

We combined the gene expression matrices of GSE45885, GSE108886, and GSE145467 and defined the new matrix as the training set; additionally, we used GSE45887 as the validation set. The “sva” package was used to remove differences between batches; additionally, we removed samples from the datasets in instances where differences between groups could not be removed (**Figure 2**) (12).

### 2.2 Differential gene analysis

Using the limma R package (13), we obtained various differentially expressed genes (DEGs) between the NOA and control groups, using a P value < 0.05 and a log2 fold-change (FC) > 1 as cut-off values. The pheatmap function was used to display the selected DEGs.

### 2.3 Consensus cluster analysis

To identify potential subclusters within the NOA samples in the training set, we clustered these samples according to their DEGs using the ConsensusClusterPlus algorithm (14). The maximum consistency score was selected as the optimal k-value, and Principal Component Analysis (PCA) was performed to verify the new classification.

### 2.4 Weighted gene co-expression network analysis

We screened the potential NOA-associated genes using the R package “WGCNA”. First, we clustered all samples and deleted genes with average expression < 0.5. Second, to better detect the strong correlation between modules, the optimal soft threshold power (β) was identified by using “pickSoftThreshold” R function. Then, we performed hierarchical clustering analysis to detect the modules, using cut-off values for minimum modularity (50) and merge height (0.25). Next, we used the “WGCNA” package to assess interaction intensity, calculate gene significance (GS) and module membership (MM), and assess the relationship between modules and clinical traits. The genes with high GS and MM screened among the gene modules of interest were considered to be key genes (15). These genes were selected for subsequent analysis. Finally, we used the “Heatmap” package to illustrate the relationship between modules and clinical traits (16).

### 2.5 Preliminary identification of signature genes and functional enrichment analysis

We defined the intersection genes derived from differential gene analysis, disease WGCNA, and cluster WGCNA as the signature genes of NOA. Using these signature genes, we performed Gene Ontology (GO), Disease Ontology (DO), and Kyoto Encyclopedia of Genes and Genomes (KEGG) enrichment analyses using the R package “clusterProfiler” (17). DO enrichment analysis was used to investigate gene-related diseases. GO enrichment analysis was used to investigate gene-related biological processes (BP), molecular functions (MF), and cellular components (CC). KEGG enrichment analysis was used to explore gene-related pathways. A P value < 0.05 was considered statistically different.

### 2.6 Biomarker identification based on five machine learning methods

We also implemented machine learning analysis using the signature genes derived from the initial screening. First, the data set was randomly divided into training and validation sets in a 7:3 ratio. Then, we used SVM, Random Forest (RF), XGBoost (XGB), and GLM algorithms to build diagnostic models for the gene expression data, using the ‘kernlab, randomForest and xgboost’ R package. The ‘caret’ and ‘DALEX’ R packages were used to optimise the model building process and to explain the relationship between input and output variables in the model, respectively. The SVM algorithm classifies data by constructing a hyperplane; additionally, it uses a regularisation term to eliminate overfitting problems from the model (18). Random survival forest is a data-driven learning algorithm that can automatically manage non-linear effects and interactions between variables (19). The XGB algorithm is an optimised model that incorporates both a linear model and a boosted tree model (20).

We then calculated the area under the receiver operating characteristic (ROC) curve (AUC) and accuracy to assess the classification ability of each model (21). Finally, we selected the algorithm with the highest AUC and fewest residuals to build the final model. The residual value is defined as the difference between the actual observations and the model estimates. A smaller residual value indicates that the model produces estimates that are in good agreement with the real data (22). We then defined the most important top 5 genes obtained with this algorithm as potential biomarkers for NOA. Subsequently, we used the “ggcorrplot” package to plot the correlation heat map between potential biomarkers (23), and searched for the start and end location of the biomarkers on chromosomes within the Ensemble Genome database (http://ensemblgenomes.org/).

### 2.7 Nomogram construction and assessment of diagnostic efficacy

To predict the incidence of NOA in infertile male patients, we used logistic regression analysis to construct a diagnostic model and visualised it using a nomogram (24). We drew a ROC curve and calculated the (AUC value using the “pROC” R package. We then used the AUC, calibration curve, and decision curve analysis (DCA) to evaluate the diagnostic efficacy. Finally, we further confirmed the differential expression and predictive reliability of the biomarkers using the validation set.

### 2.8 Regulatory mechanisms of potential biomarkers

Candidate miRNAs and lncRNAs of the potential biomarkers were identified using the miRanda, miRDB, and TargetScan databases; we then constructed a competing endogenous RNA (ceRNA) network using these candidate miRNAs and lncRNAs.

### 2.9 Immune infiltration analysis

We calculated the immune infiltration status of the testes samples using the CIBERSORT algorithm and specifically compared the expression of 22 immune cell subpopulations within the NOA and control group samples (25). Then, we further analysed the correlation between potential biomarkers and immune cells.

## 3 Results

### 3.1 Identification of DEGs in the testes of patients with NOA

**Figure 3:**
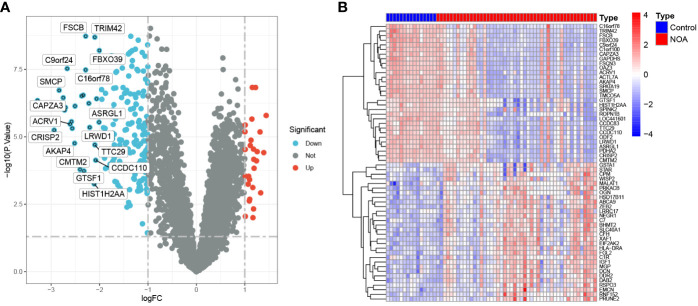
DEGs between NOA patients and control samples. **(A)** Volcano plot showing the expression levels of DEGs. **(B)** The top 30 most differentially expressed genes.

A total of 215 DEGs were identified between the NOA and control groups (27 up-regulated and 188 down-regulated genes in the NOA group), shown in the corresponding volcano plot (**Figure 3A**). The top 30 differentially expressed genes have been indicated in **Figure 3B**.

### 3.2 Weighted gene co-expression network analysis and identification of key modules

**Figure 4:**
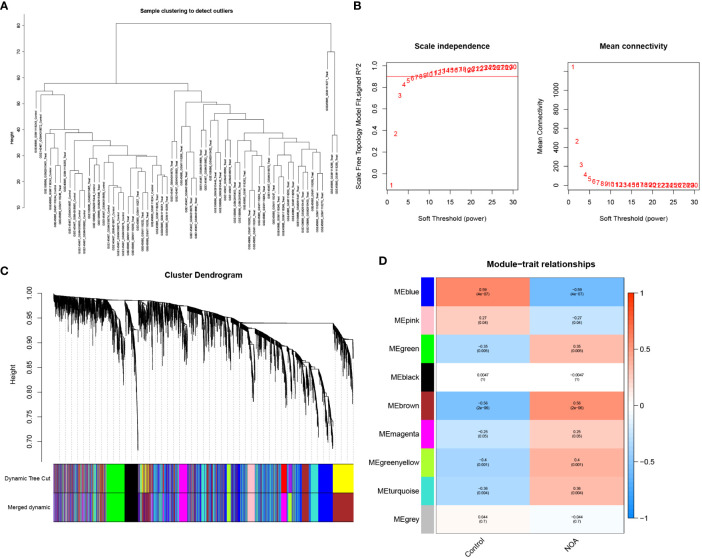
WGCNA analyses in the NOA. **(A)** The samples were classified into two clusters that were significantly distinct. All clusters were chosen for further analysis. **(B)** Soft threshold analysis suggested that gene associations were maximally consistent with the scale-free distribution and when β = 7. **(C)** Gene dendrogram obtained by average linkage hierarchical clustering. The row underneath the dendrogram shows the module assignment determined by the Dynamic Tree Cut. **(D)** Correlation between modules and NOA.

In this study, we used WGCNA to cluster highly correlated genes associated with NOA. All samples were included in the analysis after screening (**Figure 4A**). We selected 7 as the soft threshold in this study (R2 = 0.9) to construct the scale-free network (**Figure 4B**); we then screened nine co-expression modules by merging modules according to the cut-off values (**Figure 4C**). Furthermore, we analysed the module correlations and determined the highest correlation between the blue module and NOA (r=0.59, P = 4e-07) (**Figure 4D**). Finally, we considered the 1123 genes found within the MEblue module as target genes due to these genes possessing the highest correlation with NOA occurrence.

### 3.3 Unsupervised clustering and identification of key modules

**Figure 5:**
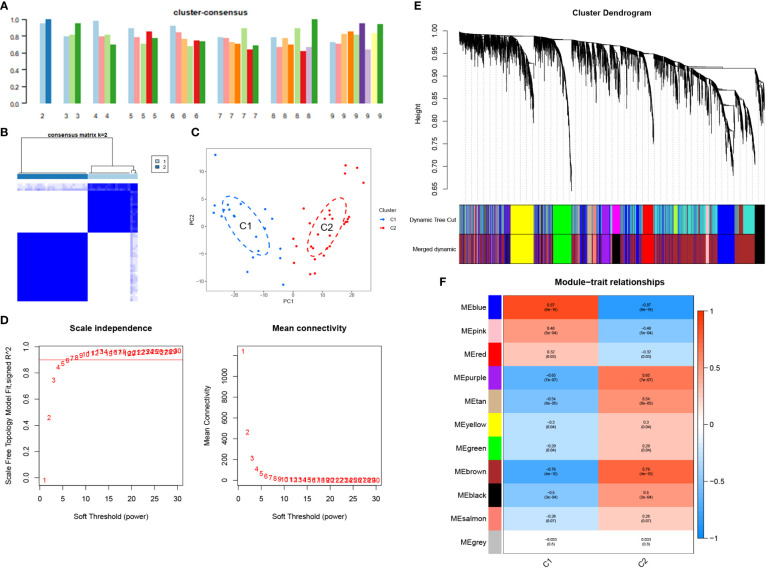
Unsupervised consensus clustering in the NOA and WGCNA analyses in the clusters. **(A)** Consistency score for k = 2 to 9. **(B)** Heatmap exhibiting the three clusters of NOA samples with k = 2. **(C)** The principal component analysis (PCA) based on the results of consensus clustering analysis. **(D)** Soft threshold analysis suggested that gene associations were maximally consistent with the scale-free distribution and when β = 6. **(E)** Modules identified by merging modules with feature factors greater than 0.25. **(F)** Correlation between modules and three clusters of NOA.

Following the removal of the control group, we performed consensus clustering on the NOA training set (48 samples). The corresponding results demonstrated that the classification was highly reliable and stable when k=2 (**Figures 5A, B**). Further, the PCA results confirmed a clear distinction between the two groups (**Figure 5C**). Finally, we divided the NOA samples into cluster 1 (C1, n=20) and cluster 2 (C2, n=28).

We also used WGCNA to cluster genes that were strongly associated with NOA classification. Using a soft threshold of 6 (R2 = 0.9), 11 co-expression modules were screened (**Figures 5D, E**). Overall, the blue module was determined to possess the highest correlation with NOA classifications (r =0.87, P = 6e-16) (**Figure 5F**). Therefore, we considered the 1027 genes in the MEblue module to be target genes.

### 3.4 Preliminary identification of signature genes and enrichment analysis

**Figure 6:**
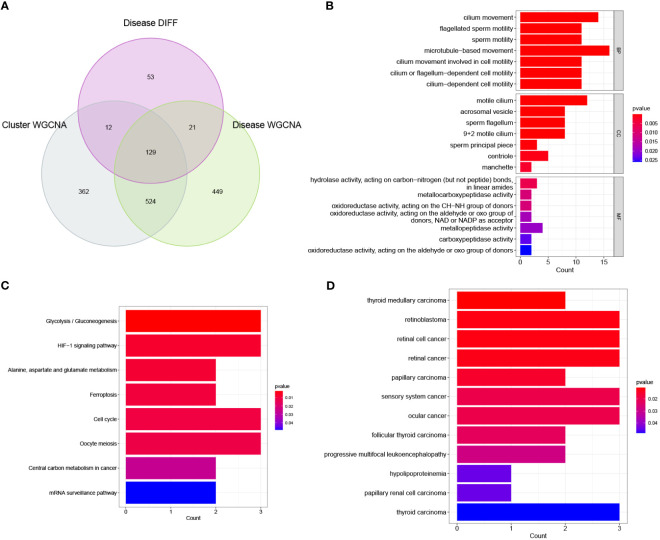
Initial identification of significant genes for NOA. **(A)** Intersection of disease WGCNA, cluster WGCNA and differential expression analysis was displayed in a Venn diagram. **(B)** Enrichment analysis of significant genes using Gene Ontology (GO). BP, biological process; CC, cellular component; MF, molecular function. **(C)** Enrichment analysis of significant genes using KEGG. **(D)** Enrichment analysis of significant genes using Disease Ontology (DO).

The 129 overlapping genes in a Venn diagram were used as signature genes (**Figure 6A**). We further performed GO, KEGG, and DO enrichment analyses to assess the potential biological functions associated with these signature genes. The GO analysis results indicated that the signature genes were primarily related to microtubule-based and cilium movement. The KEGG analysis results demonstrated that the signature genes were enriched in several pathways including in glycolysis/gluconeogenesis, the cell cycle, and the HIF-1 signalling pathway. Finally, the DO analysis results showed that these signature genes were enriched in diseases such as retinoblastoma, retinal cell cancer, and retinal cancer. The corresponding results of these enrichment analyses are shown in **Figures 6B–D**.

### 3.5 Identification of potential biomarkers

**Figure 7:**
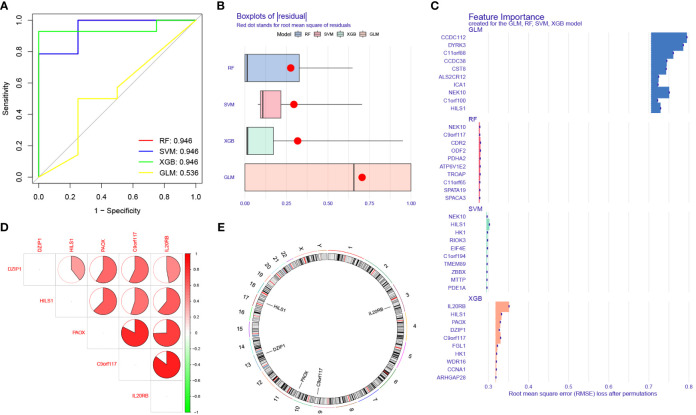
Identification of potential biomarkers for NOA based on machine learning (ML) algorithms. **(A)** Comparison of the AUC of the models with different ML classification algorithms. **(B)** Comparison of the residual of the models. **(C)** The most important top 10 genes selected by different ML classification algorithms. **(D)** Location on chromosome of the top 5 most important genes selected by XGB. The correlation network of significant genes. The darker the color of the edge, the stronger the correlation. **(E)** The location of the biomarkers on chromosomes.

We used five machine learning classification methods further screen the previously identified marker genes. Among the five machine learning models, RF, SVM, and XGB all produced an excellent AUC value (0.946) within the training sets (**Figure 7A**). We further calculated the residual values of the models using the validation sets; the residual value was determined to be the lowest in the XGB model (**Figure 7B**). Finally, we defined the 5 most significant genes (IL20RB, C9orf117, HILS1, PAOX, and DZIP1), which were identified, by XGB, as potential biomarkers (**Figure 7C**).

In this study, we also performed intergenic correlation analysis. There was a strong positive correlation observed between IL20RB and C9orf117, and between PAOX and C9orf117. There were also varying degrees of positive correlation effects between other biomarkers (**Figure 7D**). Additionally, we calculated the position of these biomarkers on chromosomes: IL20RB was determined to be on chromosome 3, C9orf117 on chromosome 9, PAOX on chromosome 10, DZIP1 on chromosome 13, and HILS1 on chromosome 17 (**Figure 7E**). The specific start and end positions of these genes are shown in **Table 2**.

**Table 2:** Location of biomarkers on chromosomes.

Gene	Chromosome	Chrom Start	Chrom End
IL20RB	chr3	136946230	137011085
C9orf117	chr9	127706988	127716002
PAOX	chr10	133379261	133391694
DZIP1	chr13	95578202	95644706
HILS1	chr17	50171428	50181255

### 3.6 Evaluation of biomarker expression levels

**Figure 8:**
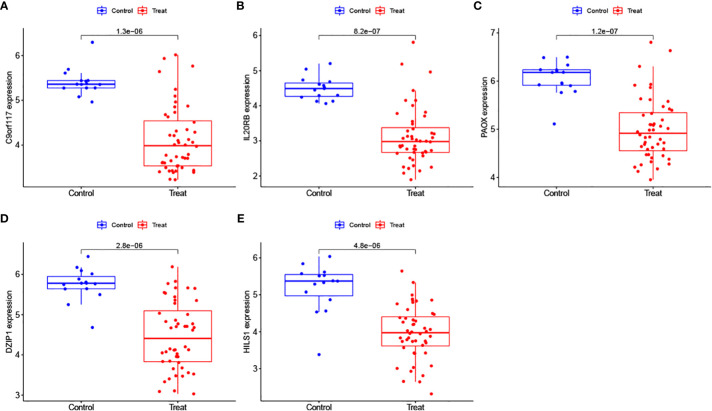
**(A–E)** Evaluation of the expression levels of the biomarkers in the training set.

**Figure 9:**
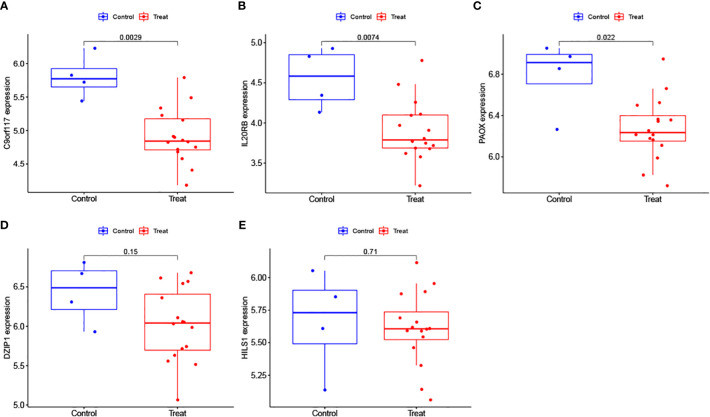
**(A–E)** Evaluation of the expression levels of the biomarkers in the validation.

We further analysed the expression levels of the 5 biomarkers in the NOA patients. The corresponding results indicated that the expression of all biomarkers was downregulated in NOA patients compared to that in the control samples (**Figure 8**). These results were similar in the validation set; although DZIP1 and HILS1 expression were not statistically different between the NOA and control groups, they also exhibited a trend towards relatively low expression in NOA patients (**Figure 9**).

### 3.7 Nomogram construction and verification

**Figure 10:**
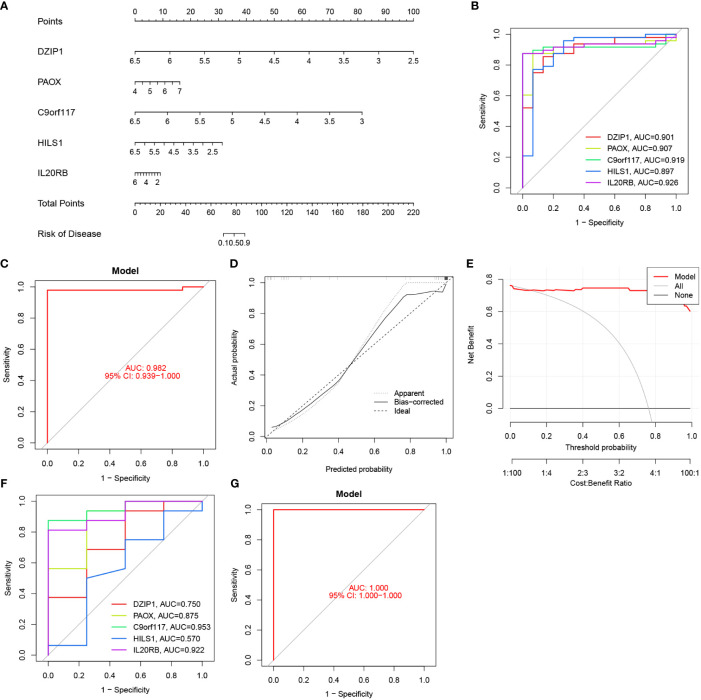
Nomogram construction and the diagnostic value evaluation. **(A)** The visible nomogram for diagnosing NOA. **(B)** The ROC curve of each candidate gene in the training set. **(C)** The ROC curve of diagnostic model in the training set. **(D)** Calibration curve. **(E)** DCA for the diagnostic model. **(F)** The ROC curve of each candidate gene in the validation set. **(G)** The ROC curve of diagnostic model in the validation set.

We used logistic regression to construct a diagnostic model based on the expressions of potential biomarkers from the NOA training set. This model has been visualised as a nomogram in **Figure 10A**. ROC analysis was also performed to compare the predictive accuracy of the model. We observed that our model was optimal, with the highest AUC value of up to 0.982, compared to other single biomarker models (**Figures 10B, C**). In addition, DCA and calibration curves indicated that this model had excellent predictive ability (**Figures 10D, E**). Finally, these results were verified using the validation set (**Figures 10F, G**).

### 3.8 Regulatory mechanisms of potential biomarkers

**Figure 11:**
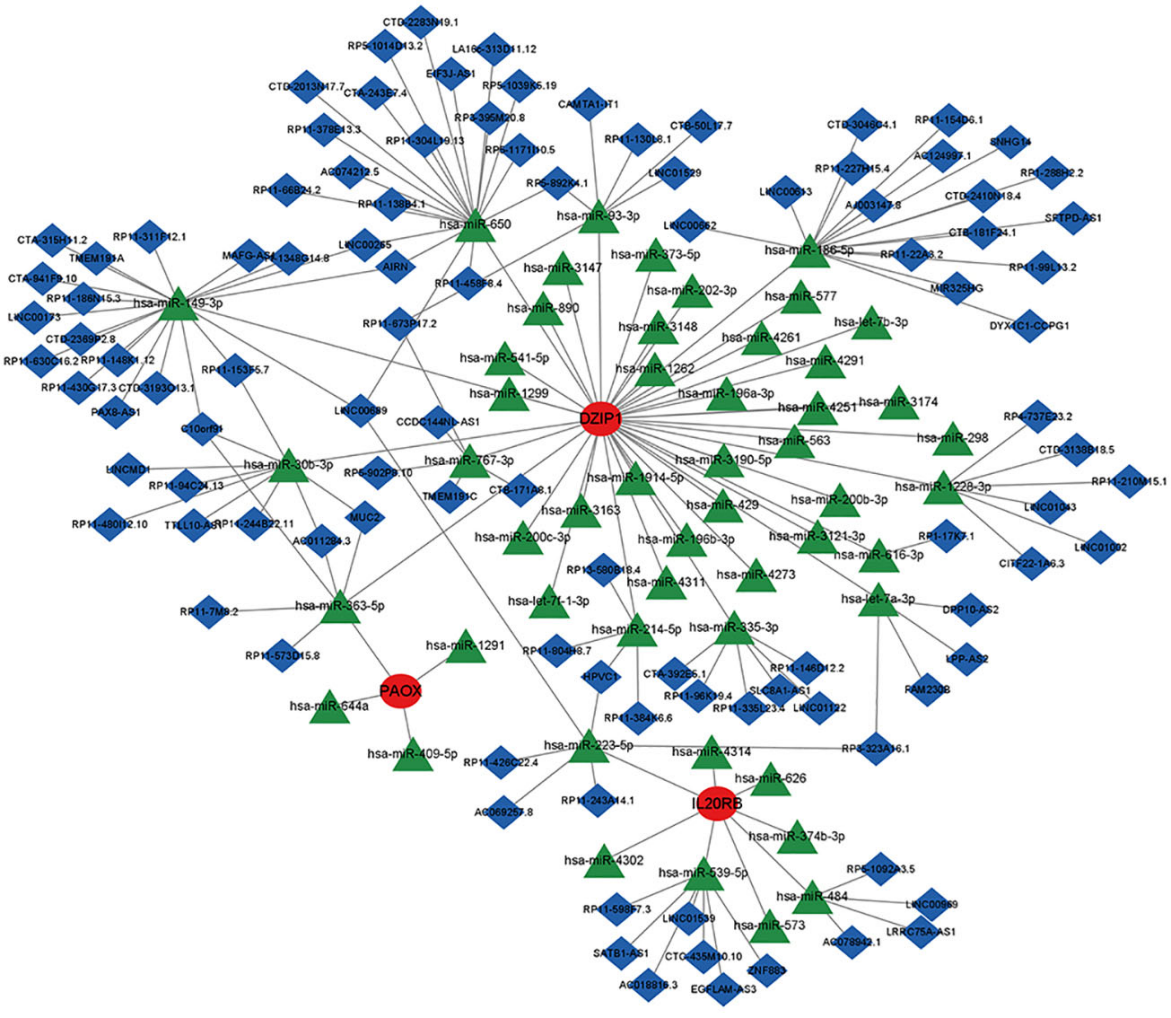
The ceRNA network of potential biomarkers. Red circles indicate potential biomarkers, green diamonds indicate miRNAs, and blue inverted cones indicate lncRNAs.

We predicted the corresponding miRNAs and lncRNAs that target the NOA-associated biomarkers using miRanda, miRDB, and TargetScan databases. To ensure accurate results, we defined the intersection of the results predicted by these three softwares as the final ceRNA prediction, which was used to construct a ceRNA network (**Figure 11**). DZIP1 and PAOX could both be regulated by hsa-miR-363-5p; additionally, DZIP1 and IL20RB could both be regulated by HPVC1 and RP3-323A16.1. Interestingly, no miRNAs or lncRNAs were predicted to target HILS1 and C9orf117.

### 3.9 Evaluation of immune cell infiltration

**Figure 12:**
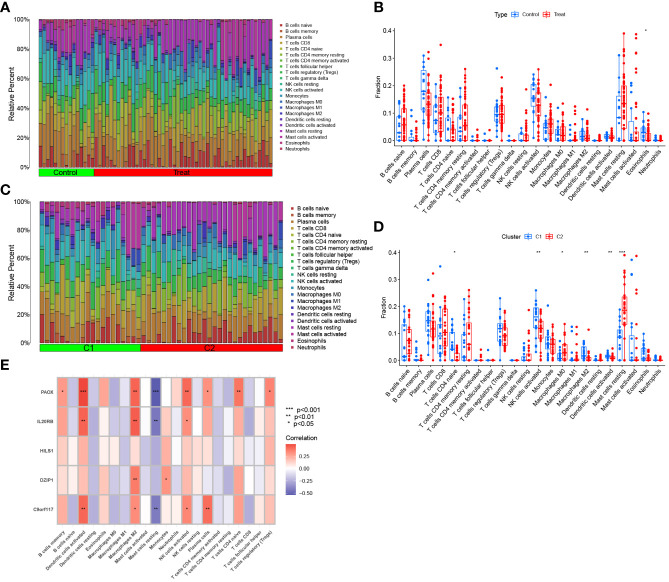
Immune cell infiltration analysis. **(A)** The proportion of 22 kinds of immune cells in different samples between NOA and control visualised from the barplot. **(B)** Comparison regarding the proportion of different immune cells between NOA and control groups visualised by the vioplot. **(C)** Immune cells in two clusters of NOA visualised from the barplot.**(D)** Comparison regarding the proportion of different immune cells in two clusters of NOA groups visualised by the vioplot. **(E)** Correlation between hub genes and 22 kinds of immune cells. *p < 0.05, **p < 0.01, ***p < 0.001.

For the NOA and control groups, the different proportions of 22 immune cell types are shown in **Figure 12A**. The analysis of immune cell infiltration demonstrated that NOA patients possessed lower levels of eosinophils (**Figure 12B**). For the C1 and C2 groups, the proportion of immune cells is shown in **Figure 12C**. The corresponding analysis indicated that, compared to patients in the C2 group, patients in the C1 group had higher levels of CD4 naive T cells, activated NK cells, M2 macrophages, and activated dendritic cells, but lower levels of M0 macrophages and resting mast cells (**Figure 12D**).

We further analysed the correlation between biomarkers and immune cells. The expression of PAOX, IL20RB, and C9orf117 were all positively correlated with infiltration by activated dendritic cells, activated M2 macrophages, and NK cells; in contrast, the expression of these genes was negatively correlated with infiltration by resting mast cells. Additionally, DZIP1 expression was also positively correlated with infiltration by monocytes and M2 macrophages. Interestingly, there was no significant correlation between HILS1 expression and immune cell distribution (**Figure 12E**).

## 4 Discussion

Non-obstructive azoospermia is the most severe and intractable form of common cause of male infertility. The process of NOA onset and development is extremely genetically complex, and is primarily caused by the dysfunction of numerous male reproductive genes and their associated regulatory signals (26, 27). Although assisted reproductive technologies can allow some patients with male infertility to have offspring, the presence of abnormal chromatin or gene defects in such patients often results in pregnancy failure or the corresponding inheritance of defective genes in the next generation (28). In addition, azoospermia not only affects fertility but is also associated with a high incidence of other diseases, such as cancer (29). Therefore, it is of great clinical value to clarify the signature genes of NOA-related differential expression to improve clinical fertility genetic counselling, eugenics, and targeted gene therapy.

In this study, gene expression profiles of NOA samples from the GEO database were assessed and obtained 15 normal spermatogenic male and 48 NOA patient samples. Initially, 215 DEGs between control and NOA samples were identified. To improve the credibility of these results and to avoid overfitting, machine learning PCA was used to classify the NOA samples, which were finally divided into two clusters. WGCNA has been successfully applied in prior studies to evaluate the association between gene modules and clinical traits; this allowed us to identify key genes associated with specific traits (6, 30, 31). Thus, using WGCNA, 1123 genes associated with NOA and 1027 genes associated with NOA classification were identified. Finally, we obtained 129 provisional overlapping genes, which were predominantly related to microtubule-based and cilium movement. Additionally, these genes were enriched in various pathways, such as glycolysis, the cell cycle, and HIF-1 signalling. We, therefore, speculated that these genes are involved in the formation of the biological structure of the flagellum; additionally, aberrant expression of these genes may result in the inability of sperm to correctly assemble.

Subsequently, potential biomarkers were identified using 5 different machine learning algorithms. Machine learning, a multidisciplinary field that has emerged in recent years, has played an important role in all aspects of medicine (32–34). Finally, IL20RB, C9orf117, HILS1, PAOX, and DZIP1 were identified as potential biomarkers using XGB algorithms that had the lowest residual value.

In general, most of the biomarkers identified in this study are relevant to reproduction. C9orf117 plays an important role in mammalian spermatogenesis, which was considered to be part of a novel mechanism that acts specifically in developing mammalian spermatozoa to ensure the formation of a single ultrastructurally correct flagellar axoneme and of a functional midpiece. Weidemann et al. determined that C9orf117 knockout mice appeared normal, but homozygous males were infertile. Overall, their study showed that C9orf117 is specifically required for flagellum morphogenesis (35). HILS1, a spermatid-specific linker histone H1-like protein, is involved in chromatin remodelling pathways during mammalian spermiogenesis, such as nuclear condensation and genetic regulation of haploid germ cell differentiation (36, 37). The low expression of HILS1 may lead to the inability to remodel sperm Chromatin, which may affect spermatogenesis (38, 39). Piotr et al. evaluated the HILS1 transcript levels in spermatozoa isolated from normozoospermic and asthenozoospermic men. Results suggested significantly lower levels of HILS1 transcripts in spermatozoa from asthenozoospermic men compared to normozoospermic men (40). This is consistent with the results obtained in this study. DZIP1 encodes a DAZ (a protein deleted in azoospermia) interacting protein, there is strong evidence that the DAZ and a closely related homolog, DAZL (DAZ-like), are required in early germ cell development to maintain initial germ cell populations (41). In addition, Lv et al. found that DZIP1 deficiency would lead to dysfunction of sperm centrioles, resulting in loss of flagella and induction of asthenoteratospermia with severe MMAF (42). In this study, the expression level of DZIP1 in NOA group was much lower than in the control group, which is consistent with the results of previous studies. PAOX was considered to be related to male yellow cattle infertility. The upregulation of PAOX may be associated with toxicity and apoptosis resistance in cattleyak (43, 44). But there is still a lack of relevant evidence on the impact of PAOX on human infertility. In addition, IL20RB, interleukin 20 receptor subunit beta, has been found to play an important role in clear cell renal cell carcinoma, while its role in reproduction remains unclear (45, 46).

As a class of small non-coding RNA molecules, miRNAs have been implicated in many biological processes, including the regulation of cell differentiation, proliferation, and death (47–49). Therefore, we investigated the regulatory mechanisms of the potential biomarkers identified in this study and determined that hsa-miR-363-5p could regulate DZIP1 and PAOX expression, and RP3-323A16.1 could regulate DZIP1 and IL20RB expression. These results are expected to shed new light on the pathogenesis and treatment of NOA.

Inflammatory diseases of the testes are major factors that cause abnormal spermatogenesis (50–52). The pathogenic mechanism of this abnormal spermatogenesis may be associated with the infiltration of immune cells and the release of various cytokines at exceptionally high levels, resulting in damage to the blood-testis barrier and the seminiferous epithelium (53, 54). In this study, eosinophils cells were determined to be enriched in control samples compared to NOA samples. We also determined that CD4 naïve T cells, activated NK cells, M2 macrophages, and dendritic cells were enriched in C1 samples, whereas M0 macrophages and resting mast cells were enriched in C2 samples. Thus, novel treatment options could be developed according to these differentially expressed immune cells.

Nonetheless, although the identified NOA-related signature expression genes were systematically analysed based on bioinformatics, we acknowledge the primary limitation of this study is the lack of external data validation. Therefore, we aim to expand the study sample size in the future and perform basic experimental validation using high-throughput sequencing technology.

## 5 Conclusion

In conclusion, by using WGCNA and machine learning algorithms, IL20RB, C9orf117, HILS1, PAOX, and DZIP1 were identified as potential therapeutic targets for NOA; additionally, we constructed a classification system for diagnostic prediction based on these five biomarkers. The regulatory mechanisms and chromosomal analysis of these genes were also revealed, providing a better understanding of their roles in NOA. In addition, our results suggested that functional changes in immune cells may play an important role in the occurrence of NOA. Therefore, our study may provide new insights into the management and treatment of patients with NOA.

## Data availability statement

The original contributions presented in the study are included in the article/**Supplementary Material**. Further inquiries can be directed to the corresponding author.

## Ethics statement

Ethical approval was not required for the study involving humans in accordance with the local legislation and institutional requirements. Written informed consent to participate in this study was not required from the participants or the participants’ legal guardians/next of kin in accordance with the national legislation and the institutional requirements.

## Author contributions

Conceptualisation: QS. Data curation: QS and QT. Formal analysis: QT. Funding acquisition: QS and QT. Methodology: QS. Supervision: QT and LW. Validation: LW and KW. Writing-original draft: KW. Writing-review and editing: KW and TJ. All authors contributed to the article and approved the submitted version.
